# Changes in Dental Biofilm Proteins’ Secondary Structure in Groups of People with Different Cariogenic Situations in the Oral Cavity and Using Medications by Means of Synchrotron FTIR-Microspectroscopy

**DOI:** 10.3390/ijms242015324

**Published:** 2023-10-18

**Authors:** Pavel Seredin, Dmitry Goloshchapov, Vladimir Kashkarov, Anatoly Lukin, Yaroslav Peshkov, Ivan Ippolitov, Yuri Ippolitov, Tatiana Litvinova, Jitraporn Vongsvivut, Boknam Chae, Raul O. Freitas

**Affiliations:** 1 Department of Solid-State Physics and Nanostructures, Voronezh State University, 394018 Voronezh, Russia; 2 Department of Pediatric Dentistry with Orthodontia, Voronezh State Medical University, 394006 Voronezh, Russia; 3 Computational Semasiology Laboratory, Voronezh State Pedagogical University, 394043 Voronezh, Russia; 4 Australian Synchrotron (Synchrotron Light Source Australia Pty LTD), Clayton, VIC 3168, Australia; jitrapov@ansto.gov.au; 5 Pohang Accelerator Laboratory, Beamline Research Division, Pohang 37673, Republic of Korea; 6 Brazilian Synchrotron Light Laboratory (LNLS), Brazilian Center for Research in Energy and Materials (CNPEM), Campinas 13083-970, Brazil

**Keywords:** dental biofilm, caries, medicinal agent, secondary structure of proteins, synchrotron FTIR-microspectroscopy

## Abstract

This work unveils the idea that the cariogenic status of the oral cavity (the presence of active caries lesions) can be predicted via a lineshape analysis of the infrared spectral signatures of the secondary structure of proteins in dental biofilms. These spectral signatures that work as natural markers also show strong sensitivity to the application in patients of a so-called modulator—a medicinal agent (a pelleted mineral complex with calcium glycerophosphate). For the first time, according to our knowledge, in terms of deconvolution of the complete spectral profile of the amide I and amide II bands, significant intra- and intergroup differences were determined in the secondary structure of proteins in the dental biofilm of patients with a healthy oral cavity and with a carious pathology. This allowed to conduct a mathematical assessment of the spectral shifts in proteins’ secondary structure in connection with the cariogenic situation in the oral cavity and with an external modulation. It was shown that only for the component *parallel β-strands* in the amide profile of the biofilm, a statistically significant (*p* < 0.05) change in its percentage weight (composition) was registered in a cariogenic situation (presence of active caries lesions). Note that no significant differences were detected in a normal situation (control) and in the presence of a carious pathology before and after the application of the modulator. The change in the frequency and percentage weight of *parallel β-strands* in the spectra of dental biofilms proved to be the result of the presence of cariogenic mutans streptococci in the film as well as of the products of their metabolism—glucan polymers. We foresee that the results presented here can inherently provide the basis for the infrared spectral diagnosis of changes (shifts) in the oral microbiome driven by the development of the carious process in the oral cavity as well as for the choice of optimal therapeutic treatments of caries based on microbiome-directed prevention measures.

## 1. Introduction

Dental caries, possibly one of the most widespread diseases globally and a significant concern for the healthcare system, has a considerable impact on people’s quality of life [1,2]. This is evident from the consistent rise in restorative dental fillings, which are not only part of the treatment process for tooth decay but also indicative of a complex relationship between caries and a number of systemic diseases [3]. The modern hypothesis regarding caries development attributes this disease to the loss of mineral components in hard dental tissue [4]. This loss is caused by changes in the composition and function of the dental biofilm and biological fluids in the oral cavity (oral and gingival crevicular fluids) driven by changes in the microbiome [5,6,7]. It should be noted that the state of the dental biofilm influences the development not only of caries but also of conditions such as periodontal disease, infections of the oral cavity, tooth demineralization, and so on [8]. As a result, leading scientific groups are not only studying variations in biofilm composition in the normal state and under the development of various diseases but also exploring the evolution of its molecular structure under different external influences (for example, when using exo-/endogenous methods of prevention and pelleted medicinal agents). Advanced studies in this domain where the processes proceeding in the oral cavity are considered at molecular level, make it possible to reasonably assert that the transition to a personalized dental service requires the molecular identification of the changes (shifts) in the composition of the biofilm (oral microbiome), which is directly connected with the organic–mineral matrix of teeth [6,9,10,11,12]. Depending on the nature of the observed pathology, either the appearance of some specific compounds (produced by the microbiome) or the transformation of the functional units composing the biofilm can be a marker of the dental health state. In the first case, the attention of researchers can be directed at the search of characteristic proteins [13,14,15], esters [16,17], thiacyanates [10,18,19], anti-inflammatory cytokines [20,21,22], the products of the bacterial microflora and their metabolites [23]. In the second case, the presence or absence of certain conformational features in the configuration of the protein biofilm fraction [24,25] is evaluated, keeping in mind that it participates in the processes of formation, transport, exchange, mineralization of the organs in the oral cavity.

The detection and identification of changes (shifts) in the dental biofilm in the initial stage of caries development prove to be a non-simple task but can be successfully achieved using spectroscopic methods for molecular identification. Thus, methods of vibrational FTIR-microspectroscopy allow obtaining unique information about molecular transformations proceeding not only in the primary but also in the secondary structure of the protein fraction of the biofilm [26,27,28]. In particular, the analysis of the secondary structure by the FTIR technique allows separating specific structural features in spite of certain similarities of the proteins composing the biofilm [29,30,31,32]. Moreover, the determination of the contribution of *α*, *β*, *R-coil* and other components to proteins’ secondary structure makes it possible to reveal changes in proteins’ structure under the effect of certain agents or factors [29,30,33]. A comparison of the results of biochemical analyses and FTIR studies of the conformational changes in the secondary structure of proteins confirmed the validity of the latter [24,34,35].

It should be noted that the study of proteins’ secondary structure by the FTIR technique requires performing a certain mathematical processing of the spectral data, namely, the deconvolution of the amide band profile into its components. This is a rather complex experimental methodological task [30,31,33,35,36,37]. Since the FTIR technique is very sensitive to a number of factors, such as water content, presence of CO_2_, very low amount of the analyte and so on, the inclusion of a microscope and a synchrotron excitation source into the gauging scheme at some stages of the scientific investigation allows not only obtaining the optimal signal-to-noise ratio but also studying the molecular changes in the microbiome with high spatial and spectral resolution [26,38].

In spite of the fact that the reasons of shifts in the oral microbiome for different diseases of the oral cavity are the object of intensive studies [7,27,39], the determination of the biofilm microbial composition is sometimes insufficient to reveal its relationship with caries development [5,7,39]. Therefore, it is necessary to analyze the changes in the secondary structure and conformation of the protein fraction in the dental biofilm of people with active caries. This issue has been insufficiently addressed in the literature and demands additional efforts.

Therefore, the aim of our work was to compare changes in the secondary structure of biofilm proteins in patients with different caries activity, including patients using medicinal agents for caries prevention and for promoting the mineralization of dental enamel (external modulation), with the use of synchrotron FTIR-microspectroscopy.

## 2. Experimental Results

The design of the experiments in this study was similar to that described in our previous work, where the molecular composition of the dental biofilm in patients undergoing exo- and endogenous caries prevention was analyzed [26]. A preliminary exam of the obtained results showed that the IR spectra of the dental biofilm of the group of participants without caries (normal state) and of the patients with active caries were in excellent agreement with the results obtained in the previous investigations [26,27,28]. The analysis demonstrated that in certain experimental conditions, the IR spectra of the samples included one and the same set of absorption bands corresponding to the characteristic molecular bonds inherent in the dental biofilm. Moreover, the spectra of the samples in different experimental conditions (without and with the use of the modulator) and in the absence or presence of active carious lesions (normal state/pathology) differed only insignificantly from one another, which was probably due to the individual features of the subjects participating in the experiment. Therefore, for convenience, the IR spectra representing the average spectra of samples without and with the modulator will be presented in this work.

**Figure 1:**
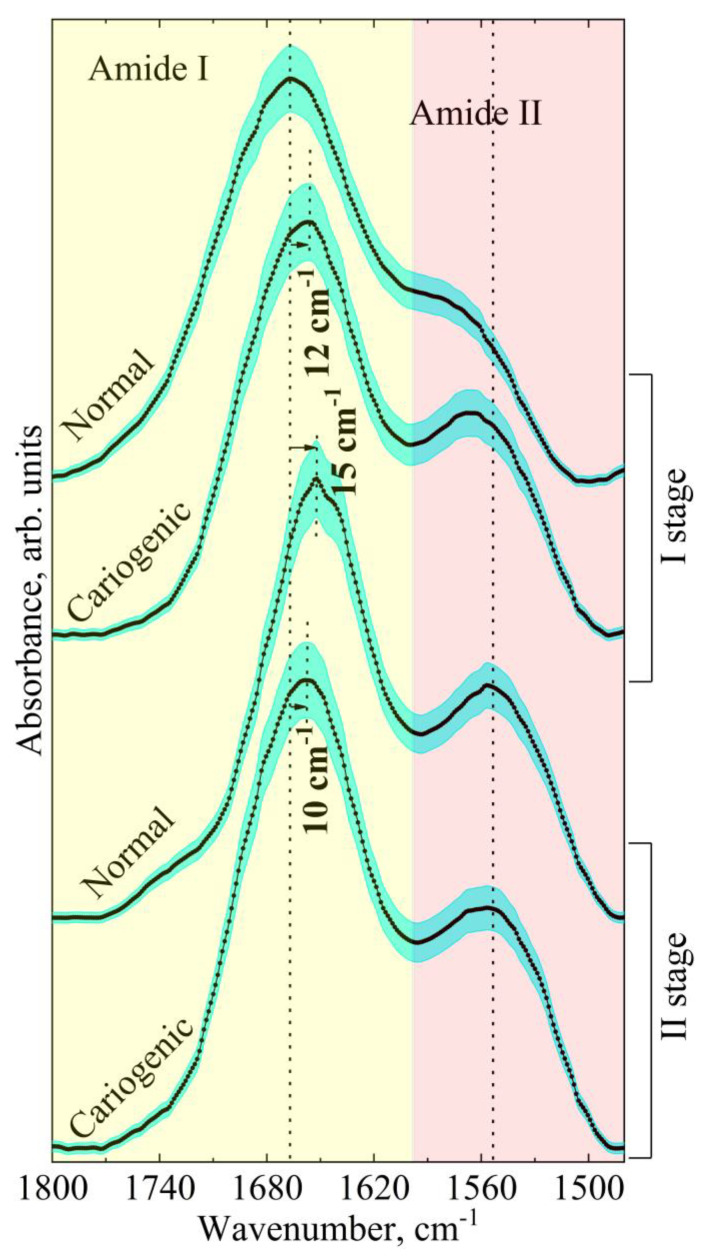
Total spectral profile of the amide I and amide II bands in averaged IR spectra of dental biofilm for patients with a normal enamel and a carious pathology before (I experimental stage) and after (II experimental stage) the modulator effect (use of tablets with a mineral complex involving calcium glycerophosphate). The averaged spectra are represented with the error deviation bar.

Previously, it was demonstrated that the most informative way to estimate changes in the secondary structure of proteins was to use the amide bands amide I or amide II, which are very sensitive to their surrounding conformation [29,30,33,36,40]. Moreover, the amide bands are located in that part of the spectral range which rather often does not include the absorption spectra of phospholipids and even more complex molecular mixtures including those present in bacterial films [27,39]. Therefore, in our work, we limited our analysis to a spectral range of 1800–1480 cm^−1^, where the complete spectral profile of amide I and amide II is located. Figure 1 illustrates the shape of the amide profile in the IR spectra of biofilms of the two groups of participants in the two experimental conditions examined. The spectra in Figure 1 were selected in such a way as to clearly show both the presence of active carious lesions in the patients and the use of the modulating factor (tablets containing a mineral complex with glycerophosphate). A comparative analysis showed that in the presence of caries activity (normal state—pathology), not only the ratio of the intensities and the half-width of the amide I and amide II peaks changed, but also their frequency.

Note that a considerable low-frequency shift of the amide I peak was detected in the spectra of biofilm sampled from people with active caries (second group) relative to the position of that detected for the patients without caries (first group) (Figure 1). At the same time, the application of the prevention medication (tablets with a mineral complex based on calcium glycerophosphate) eliminated the difference in the position of the amide I band in the spectra of both groups due to the low-frequency shift of this vibration band in the IR spectra of the first group. At the same time, the position of the amide I band in the biofilm spectra of the patients of the second group (with active caries) in the presence of the modulating factor (tablets) varied insignificantly. Similar transformations of the spectral profile of the biofilms were registered for the amide II band (Figure 1).

The transformation of the spectral profile of the amide I and amide II bands was considerably due to changes in the secondary structure of proteins contained in the biofilm [39], as well as to microbiotic products associated with the presence of active caries lesions in the oral cavity of the patients [8,41]. Therefore, in order to determine the effect of active caries, as well as of exogenous modulators, the secondary structure of the protein net in the biofilm was studied and visualized on the basis of the deconvolution of the total spectral profile of the amide I and amide II bands in the IR spectra. The spectral profiles were analyzed in the range of 1800–1480 cm^−1^ with the use of program packages for data processing and simulation of non-linear curves, i.e., Fityk (version 1.3.1) [42] and Origin Lab (b9.5.0.193) [33]. Detailed information for data processing and simulation is presented in the Methods section.

**Figure 2:**
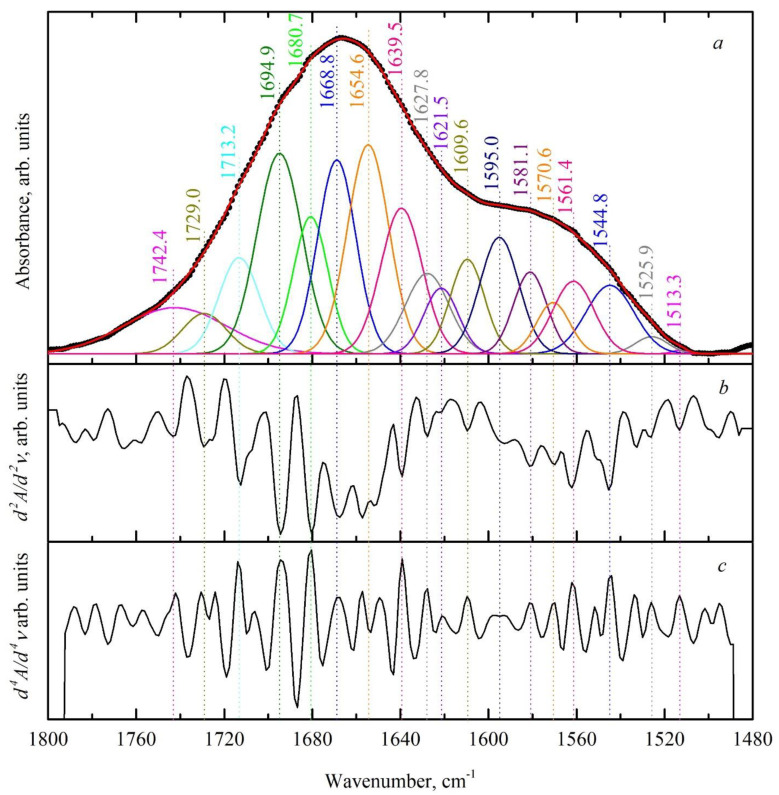
Experimental and simulated FTIR spectral profiles in the absorption range of the amide I and amide II bands for a typical sample of dental biofilm, as well as second and fourth derivatives of the experimental spectrum. (**a**) Experimental and simulated FTIR spectral profiles; (**b**) second derivative; (**c**) fourth derivative.

Experimental and simulated FTIR spectra of the dental biofilm in the range of 1800–1480 cm^−1^ are presented in Figure 2; they were obtained using the technique of deconvolution along with the second and fourth derivatives of the experimental spectrum.

**Figure 3:**
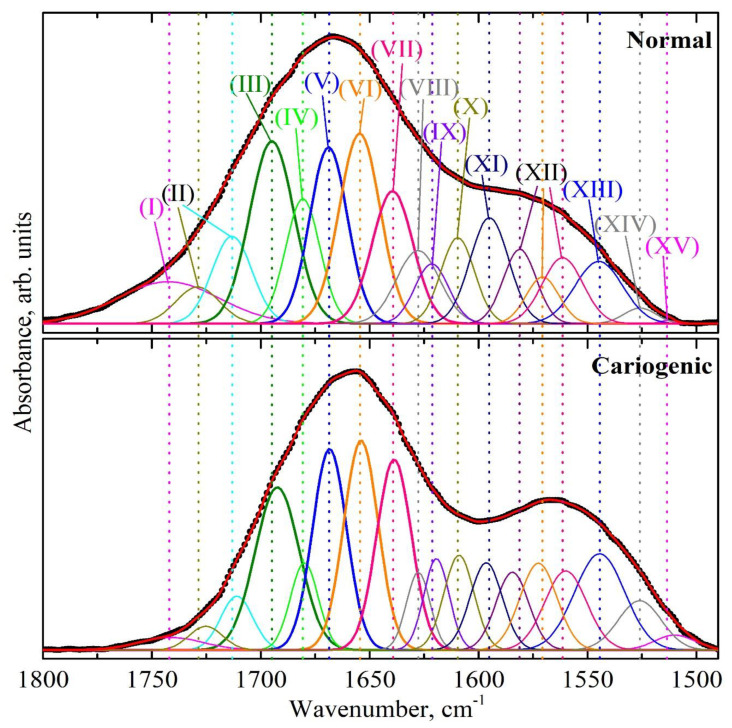
Results of the deconvolution of the spectral profile in the range of the amide I and amide II bands for typical samples of biofilm from patients with a normal enamel (top) and with carious pathology (bottom) in the first stage of the experiment without the modulator (tablets with a mineral complex involving calcium glycerophosphate). Absorption bands in the IR range were associated with the following components of proteins’ secondary structure: (I) ν(C=O), acid esters; (II) ν(C=O), lipid esters; (III) *anti-parallel β-strands*, *β-turn;* (IV) *parallel β-sheet*, *β-turn*; (V) *β-turn*; (VI) *α-helix*; (VII) irregular structure, triple helix; (VIII) *parallel β-strands*; (IX) *β-sheet, β-turn*; (X) amino acid side chain, intermolecular *β-sheet*; (XI) δ(NH_2_), amine; (XII) δ(N–H); (XIII) *α-helix*, δ(N–H); (XIV) *β-sheet*, δ_s_(NH^+^_3_); (XV) ν(CN), δ(CH), δ(NH).

**Figure 4:**
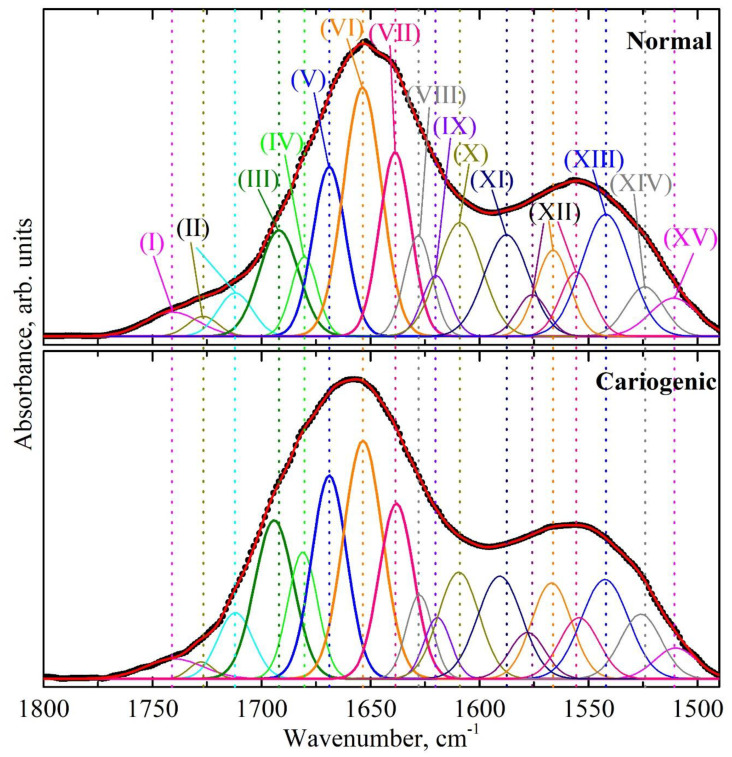
Results of the deconvolution of the spectral profile in the range of the amide I and amide II bands for typical samples of biofilm from patients with a normal enamel (top) and with carious pathology (bottom) in the second (II) stage of the experiment in the presence of the modulator (tablets with a mineral complex involving calcium glycerophosphate). Absorption bands in the IR range were associated with the following components of proteins’ secondary structure: (I) ν(C=O), acid esters; (II) ν(C=O), lipid esters; (III) *anti-parallel β-strands*, *β-turn*; (IV) *parallel β-sheet*, *β-turn*; (V) *β-turn*; (VI) *α-helix*; (VII) irregular structure, triple helix; (VIII) *parallel β-strands*; (IX) *β-sheet*, *β-turn*; (X) amino acid side chain, intermolecular *β-sheet*; (XI) δ(NH_2_), amine; (XII) δ(N–H); (XIII) *α-helix*, δ(N–H); (XIV) *β-sheet*, δ_s_(NH^+^_3_); (XV) ν(CN), δ(CH), δ(NH).

The analysis of the results of the amide profile deconvolution into its components, was based on previous works that examined various proteins including those contained in biofilms [26,27,39,43,44]. Hence, in the infrared profile of the amide I and amide II bands, the following fundamental secondary structure elements were detected: the regular secondary structures *α-helix*, *β-sheet*, *β-turn*, amino acid side chains. A great part of the integral intensity in the spectral profile corresponded to the *α-helix* and *β-sheet* structures. Rather often, parallel and anti-parallel components were detected in the *β-sheet* structure, formed by several *β-strands*; similarly, for the *α-helix*, we distinguished ordered and disordered (tips of amino acid chains) portions [45]. Previously, it was shown that due to a low dispersion, the values of deconvolution of the *parallel β-sheet* and the disordered content of the *α-helix* rather frequently cannot be predicted satisfactorily [31]. Therefore, based on the decomposition of the spectral profile of the dental biofilm in the range of the amide I and amide II bands, the following components of proteins’ secondary structure were separated: *α-helix* (ordered part); a disordered component; *β-sheet*, with its individual components of *parallel β-strands* and *anti-parallel β-strands*; *β-turn*; and a component which was the sum of *anti-parallel β-strands* and *β-turn* (Figure 3 and Figure 4). In addition, a considerable part of peak intensity in the range of the amide I and amide II bands in the form of *β-sheet* maximums was due to amino acid side chains, i.e., to vibrations of the CN, CH, NH groups and amines, as well as of C=O bonds, belonging to lipid and acid esters [36].

The results of profile deconvolution, namely, the frequencies of the maximums (centers of gravity), full width at half maximum (FWHM) and integral intensities of the separated components, attributed to the elements of the secondary structure of proteins, are presented in Table 1. Table 1 shows the average values determined with the *t*-test. The components of the proteins’ secondary structure in dental biofilm in the IR spectral profile of the amide I and amide II bands, separated according to the results of deconvolution, are designated by roman numerals (see Table 1 and Figure 3 and Figure 4).

## 3. Discussion

Analyzing the deconvolution data, one can notice that the appearance of a cariogenic situation in the oral cavity (from a normal state to caries) resulted in a frequency shift of several components (Figure 3 and Figure 4). For example, a rather significant spectral shift by 2 cm^−1^ was observed for the modes corresponding to the bonds of ν(CN), δ(CH), δ(NH) (component XV) at ~1510 cm^−1^, δ(N–H) (component XII) at ~1560 cm^−1^, 1570 cm^−1^, 1580 cm^−1^, amine (component XI), *β-sheet + β-turn* (component IX), *anti-parallel β-strands + β-turn* (component III) and the ν(C=O) vibration attributed to lipid esters (component II). At the same time, changes in FWHM were registered as well as in the integral intensities of these maximums. In addition, changes in all these components were observed in the presence of the modulating factor, by pairwise comparison of the dental biofilm spectra for the participants of both groups before and after the administration of the tablets with a mineral complex based on calcium glycerophosphate. However, one can note that the spectral position of the components *α-helix*, *β-turn* and *β-sheet* in the secondary structure of dental biofilm proteins in samples from the I stage normal group (1654.6 cm^−1^, 1668.8 cm^−1^, 1621.6 cm^−1^), I stage caries group (1654.0 cm^−1^, 1668.4 cm^−1^, 1619.3 cm^−1^), II stage normal group (1653.7 cm^−1^, 1668.9 cm^−1^, 1621.6 cm^−1^), II stage caries group (1653.5 cm^−1^, 1668.9 cm^−1^, 1619.3 cm^−1^) remained stable, as observed with the appearance of a cariogenic situation in the oral cavity (from a normal state to caries) and when using the modulator (Figure 3 and Figure 4, Table 1).

The IR spectral profiles of different proteins (depending on their amino acid composition) [36] have been previously used to study cariogenic bacteria as well as the products of their metabolism—glucan polymers [39,41]. As it was shown in the work by Gieroba et al. [39], in the FTIR spectra of mutans streptococci, high-intensive vibrations were observed in the range of the amide I and amide II bands, i.e., at 1642–1622 cm^−1^ and 1536–1522 cm^−1^, respectively. As for glucan polymers [7,8,41,54], which are produced by *Streptococcus mutans* and play a great role in the formation of the biofilm, various studies demonstrated that the metabolism products of mutans streptococci also displayed spectral features in the range of 1620–1650 cm^−1^ [50]_._ The analysis of the experimental data obtained in our work (Figure 3, Table 1) allowed noticing the presence of spectral features in the amide profile of dental biofilm, characteristic of mutans streptococci (mainly, *Streptococcus mutans* and *Streptococcus sobrinus*) and glucan polymers ~1628 cm^−1^. This was also confirmed by the fact that in the group with active caries, an increase in the integral intensity of vibrations for the components of secondary structure attributed to mutans streptococci and glucan polymers (in the range of 1625 cm^−1^ and 1525 cm^−1^) was observed, compared with the group without active caries (normal state).

To visualize the changes in proteins’ secondary structure, some valid intragroup and intergroup differences were determined for both groups of participants in different experimental conditions, based on the changes in the spectral profiles. From a comparison of the spectral curves (Figure 3 and Figure 4), one can conclude that the total intensity of the spectral amide I and amide II bands changed depending on the experimental conditions (normal state, pathology, modulating factor). Therefore, to make a correct estimation of the shifts in the protein profiles of the biofilms, it is necessary to use, rather than the integral intensities of certain separate components of the secondary structure, their percentage weights, i.e., the intensities reduced to the total integral intensity of a complete spectral profile of the amide I and amide II band [43].

**Figure 5:**
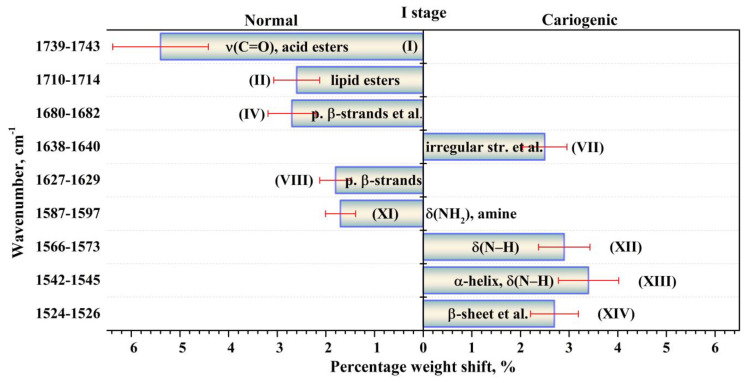
Valid (*p* < 0.05) intergroup differences in the percentages (content) of the components of the secondary structure of dental biofilm proteins between the patients in the first group (normal enamel) and those in the second one (carious enamel) in the first (I) stage of the experiment (without the use of a medicinal agent intended to promote the mineralization of the dental tissue). The intergroup differences in the percentages are represented with the error deviation bar. Roman numbers are labeled the corresponding components of proteins’ secondary structure.

**Figure 6:**
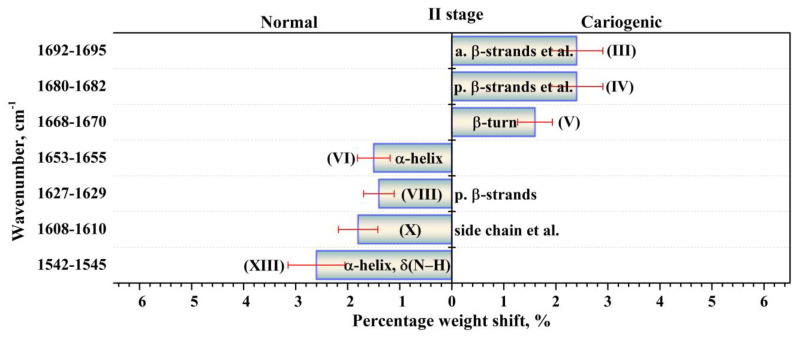
Valid (*p* < 0.05) intergroup differences in the percentages (content) of the components of the secondary structure of dental biofilm proteins between the patients in the first group (normal enamel) and those in the second one (carious enamel) in the second (II) stage of the experiment (after the use of a medicinal agent intended to promote the mineralization of the dental tissue). The intergroup differences in the percentages are represented with the error deviation bar. Roman numbers are labeled the corresponding components of proteins’ secondary structure.

**Figure 7:**
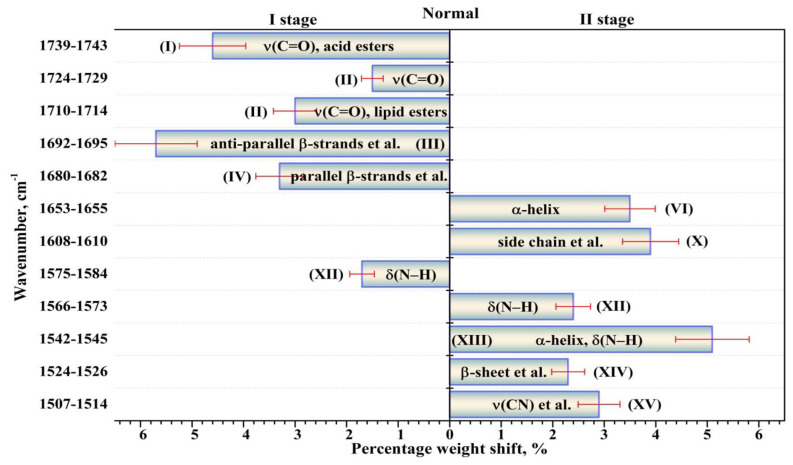
Valid (*p* < 0.05) intragroup differences in the percentages (content) of the components of the secondary structure of dental biofilm proteins before (I stage of the experiment) and after (II stage of the experiment) the use of the modulator for the group of participants with a normal cariogenic status. The intragroup differences in the percentages are represented with the error deviation bar. Roman numbers are labeled the corresponding components of proteins’ secondary structure.

**Figure 8:**
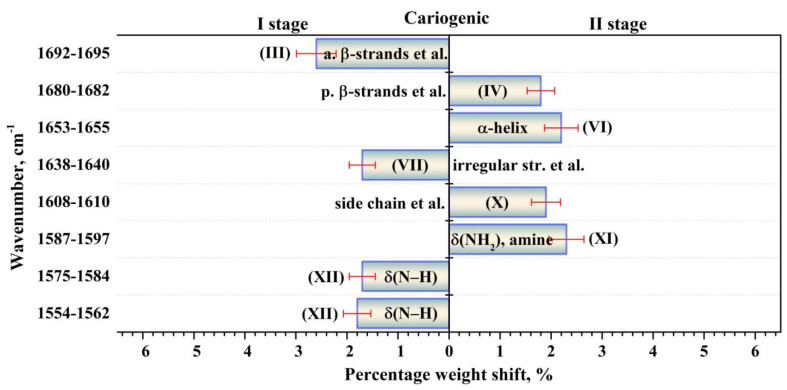
Valid (*p* < 0.05) intragroup differences in the percentages (content) of the components of the secondary structure of dental biofilm proteins before (I stage of the experiment) and after (II stage of the experiment) the use of the modulator for the group of participants with carious pathology (active caries). The intragroup differences in the percentages are represented with the error deviation bar. Roman numbers are labeled the corresponding components of proteins’ secondary structure.

The performed intragroup pairwise comparisons of the percentage weights of the components of the biofilm secondary structure in the I and II stages of the experiment for patients in the normal state and with a carious pathology made it possible to reveal the effect of the modulating factor (tablets). Duncan’s test for multiple comparisons was used. At the same time, statistically valid intergroup differences for the two groups of participants were determined applying the results of dispersion analysis with the use of a non-parametric criterion from the Kruskal–Wallis one-way analysis. To do this, we compared the percentage weights of the components of biofilm proteins’ secondary structure for the group of patients with normal enamel and for the group with active caries, as well as for the group not using the external modulator (tablets)—I stage of the experiment—and for the group using it—II stage. The revealed statistically valid intragroup and intergroup differences with *p* < 0.05 are presented in Figure 5, Figure 6, Figure 7 and Figure 8. Figure 5, Figure 6, Figure 7 and Figure 8 present the differences (change) in the percentage weights only for those components of the secondary structure that demonstrated significant differences. This clearly made it possible to estimate the shift in secondary structure depending on the presence of a cariogenic situation in the oral cavity and of an external modulation.

A comparative analysis of two sets of the components (Figure 5 and Figure 6), for which significant differences (shifts) in the percentage weights at intergroup comparison (normal state–caries) were observed, demonstrated that the use of the modulator (tablets) significantly influenced the quantitative and qualitative composition of the sets. In the I stage of the experiment (before using the modulator), statistically valid differences in the percentage weights in intergroup comparison were determined for nine components of the secondary structure (Figure 5), while after the use of the tablets in the II stage of the experiment (Figure 6), they were observed for seven components of the secondary structure. One should note that valid differences (shifts) in the percentage weights during intergroup comparison (normal state and carious pathology) for the components of *parallel β-strands + β-turn* (IV) at ~1680 cm^−1^, *parallel β-strands* (VIII) at ~1628 cm^−1^, *α-helix* + δ(N–H) (XIII) at 1544 cm^−1^ were surveyed in both stages of the experiment (before and after the use of the modulator). Moreover, the shift (difference) in percentage weight for these components (−2.7, −1.8, 3.4 for I stage and 2.4, −1.4, −2.6 for II stage) depended on the stage of the experiment. These facts indicated a simultaneous effect on these components of the secondary structure of dental biofilm proteins caused by both a cariogenic situation in the oral cavity and the modulator.

Not less interesting is the fact that the value of the shift in percentage weight for the component *parallel β-strands* determined during intergroup comparison did not change (−1.8) when using the tablets (II stage of the experiment, Figure 6) in comparison to that observed in the I stage of the experiment (−1.4) (Figure 5). It means that the change in percentage weight for the *parallel β-strands* component in the total amide spectral profile took place only under the influence of a cariogenic situation, while the use of the modulator has no impact.

A lot of substances present in medicinal agents, such as free amino acids, are known to have an effect on absorption in the range of the amide bands [55]. Our analysis of the significant changes in the percentage weights observed in the intragroup comparison between the patients in a normal state and those with carious pathology (active caries) before and after the use of the modulator confirmed this idea. First, for patients in the group without active caries (in the normal state), the use of a pelleted mineral complex with calcium glycerophosphate resulted in a change of the percentage weights for practically all the components of the secondary structure (Figure 7). At the same time, for the patients with carious pathology (active caries), significant changes in the percentage weights when using the modulator were detected only for certain components (Figure 8). One can note that significant differences in the percentage weights were not detected only for the components *β-turn* (V) near ~1669 cm^−1^, *parallel β-strands* (VIII) near ~1628 cm^−1^, *β-sheet + β-turn* (IX) near~1620 cm^−1^, either alone or together, during the intragroup comparison of patients with a normal state/carious pathology before and after the application of the modulator.

Previously, it was shown [26,56] that the calculation of coefficients corresponding to the ratio of the intensities of vibration bands associated with specific molecular groups in the oral and gingival fluids as well as in dental biofilm provided a mathematical estimation of the changes in the molecular composition of bioanalytes and also allowed screening for the development of pathologies in the oral cavity. This conclusion was based on the scientifically proved fact that biological fluids in the oral cavity, with conducting, transport and buffer functions, as well as dental biofilm contain characteristic set of ions, complexes, proteins and other molecules that may signal the development of infections in the oral cavity or pathological processes in the dental tissue [11,13].

Considering the features of the secondary structure of the protein net in the dental biofilm in the normal state, in the presence of a carious pathology, as well as after the application of the pelleted mineral complex with calcium glycerophosphate, it is possible to suggest that the ideal candidate to examine when screening for disease development is the component *parallel β-strands* (VIII) near 1628 cm^−1^. Changes in the frequency of vibrations and in the percentage weight of this component in the spectra of dental biofilm proved to be a consequence of the presence of cariogenic mutans streptococci [39] in the film as well as of the products of their metabolism—glucan polymers [39,41]. Taking into account the fact that the percentage of *α-helix* in the secondary structure of biofilm proteins remained invariable (Figure 5), an empirical calculation of $R=\frac{\alpha -helix(V)}{\beta -strands(VIII)}$ is proposed. R represents the changes in the secondary structure of the biofilm proteins observed during the development of carious pathology. Calculations demonstrated that in the presence of the modulating factor (use of the pelleted complex to promote the mineralization of the dental tissue) in subjects without carious activity (first group of patients), this ratio R was within the limits of ~2.5–2.9, while for the patients with active caries (second group), R was ~3.9–4.2, i.e., it was augmented by more than 1.5 times. Indeed, the ratio of *α-helix/β-sheet*, as it was previously demonstrated by Titus et al. [29], is a statistically valid marker of the development of inflammatory processes. In the work by Huang et al. [57], using albumin serum of the human blood as an example, it was proved that the appearance of *β-sheet* intramolecular structures was connected with the aggregation of protein molecules that had a negative effect on the functioning of these proteins. Thus, the presented empirical ratio R, determined from the analysis of the transformation in the secondary structure of the dental biofilm proteins, can be used for the rapid screening and monitoring of the development of a cariogenic situation in the oral cavity of patients, as a new precise diagnostic marker useful also for disease prevention.

One should realize that the composition and functional profiles of microbial communities considerably differ in the presence of enamel caries and dentin caries, as well as in different areas on the surface of dental enamel.

In our study, while exploring the influence of a cariogenic situation in the oral cavity of patients as well as of the modulator on the secondary structure of proteins, the work by Gieroba et al. [39], where the FTIR spectra of biofilms produced by certain *Streptococcus* strains were analyzed, was taken into account. Surely, mimicking in vitro the natural environment inhabited by communities of oral bacteria in order to perform a FTIR analysis is an awkward task. This caused limits to our work, concerning the analysis of the spectral characteristics of complex bacterial products derived from the activity of various *Streptococcus mutants* strains.

## 4. Objects and Methods of Investigation

### 4.1. Design of the Study

This study was conducted according to the guidelines of the Declaration of Helsinki and approved by the Institutional Review Board (protocol number: Pr-008.014.2021, 1 August 2021).

Fifty people participated in the study (men and women), aged 18–25 years, physically healthy, without bad habits. The patients led a standard lifestyle, eating healthy food; they did not take drugs and did not drink alcohol.

The participants were divided in two groups. The first group (*n* = 25) had no clinically discernible carious lesions of the dental tissue (ICDAS 0 [58]). The presence of active caries lesions [59] (ICDAS 1—2 [58]) was identified in participants of the second group (*n* = 25) during examination. It is worth noting that no signs of development of periodontitis or gingivitis were revealed in any of the participants.

Samples of the biofilms were taken in the morning before eating. Before that, a mechanical purification of the teeth was executed with the use of a soft toothbrush in order to remove residuals of the plaque and a preliminary gargle of the oral cavity with pure water was carried out. After 30 min, the biofilm was accurately removed from the surface of the maxillary central incisors of the patients using a sterile scalpel, without touching the gingival sulcus.

The removal of the biofilm was performed in two stages. In the first (I) stage, the biofilm was taken on the first day of the experiment. The next day, the patients began to take tablets containing a mineral complex with calcium glycerophosphate [60,61]. According to the recommendations of the producer, the participants took one tablet 3 times a day. On the fourth day, the biofilm was collected a second time. After collection, the samples of biofilm were placed into sterile containers and kept at the temperature of 4 °C before the analyses. The samples were transported to the lab and analyzed within 7 days after collection. In this study, we did not analyzed the samples in duplicate, triplicate, etc.

### 4.2. Equipment Setup and Sample Scanning

The infrared absorption spectra of the biofilm samples were surveyed using the infrared microspectroscopy (IRM) beamline (Australian synchrotron, Victoria, Australia) and the infrared spectroscopy beamline, Pohang Accelerator Laboratory (Pohang, Korea). To perform IR microspectroscopy, an IR spectrometer Bruker Vertex 80 v was used coupled to a FTIR microscope Hyperion 2000. Detection was performed with a liquid-nitrogen-cooled narrow-band mercury–cadmium–telluride MCT detector (Bruker Optik GmbH, Ettlingen, Germany). High-sensitivity spectral analysis was performed at the IMBUIA beamline of the fourth-generation storage ring Sirius, at the Brazilian Synchrotron Light Laboratory (Campinas, Brazil), using an Agilent Cary 660 FTIR microscope operating with a thermal source. All the synchrotron FTIR spectra were surveyed in the spectral range of 2000–700 cm^−1^, with spectral resolution not lower than 4 cm^−1^.

### 4.3. Data Processing

The preliminary processing of the spectral curves (spectral amid I and II bands) included a correction of the baseline based on the procedure of rubber band correction. For the approximation of the FTIR spectral profiles, Gauss functions were chosen, as they mostly correctly describe the shape of vibration modes in IR spectra [33,62]. The number of components in the secondary structure and the determination of the gravity center for the peaks were determined by finding the extreme points in the experimental spectral curves. To perform this, second and fourth derivatives were calculated [33,35]. The spectra of the second derivative were smoothed with the polynomial Savitsky–Golay function of the second order (8–10 points), while those of the fourth order derivative were smoothed with the Savitsky–Golay function of the fourth polynomial order (10–15 points). Optimization of the position and the shape of the peaks was performed with the use of the Levenberg–Marquardt Fityk algorithm [42]. The number of maximums describing the experimental spectral curve for all samples was kept invariable during the simulation procedure.

The centers of gravity for the peaks (frequencies) and full width at half maximum (FWHM) of the peaks were specified based on the analysis of the second and fourth derivatives. Bound constraints (which are also called box constraints or, more generally, inequality constraints) were specified in order that the parameters of the peaks could not go beyond the limits of the boundaries. During the simulation procedure, the positions of the peaks could change within ±2 cm^−1^. In the spectral range of 1700–1600 cm^−1^, the boundary conditions for FWHM were 5–25 cm^−1^. The low boundary was taken for the description of amide I. In turn, the upper value of the band width was most correct for the description of the amide I band, as demonstrated in the works by Carrasquillo and Stani [51,63]. The simulation procedure was halted when the best correspondence between simulated and experimental curves was achieved according to the χ-square criterion. Consequently, the required criterion of convergence and reproducibility of the simulation results was found, which ensured the unambiguity of the decomposition, which was checked on a large number of spectra.

### 4.4. Statistical Analysis

Statistical analysis of the data was performed with SPSS 29 software (SPSS Inc., Chicago, IL, USA). We used a standard *t*-test for the descriptive statistics. To reveal intergroup differences, non-parametric dispersion analysis ANOVA and Kruskal–Wallis one-way analysis were used. Statistically significant changes within the groups (intragroup differences) were determined based on the multiple-comparison test of Duncan.

## 5. Conclusions

Changes in the secondary structure of dental biofilm proteins were studied based on the deconvolution of the IR spectral profile of the amide I and amide II bands. Employing a large set of spectra, it was shown that the secondary structure of the protein net in the biofilm was affected by the presence of a cariogenic situation in the oral cavity (the presence of active caries lesions), as well as by the application of a modulator—a medicinal agent (a pelleted mineral complex with calcium glycerophosphate). In spite of the fact that both of these factors did not result in a qualitative change of the composition, the changes revealed different quantitative characteristics (changes in the frequencies, FWHM and integral intensities) of the components of dental biofilm proteins’ secondary structure.

Proceeding from the transformation of the total spectral profile of the amide I and amide II bands, valid intragroup and intergroup differences were determined in the secondary structure of the proteins in the dental biofilm for the patients with a normal enamel and those with carious pathology in the oral cavity, also after the administration of the tableted mineral complex with calcium glycerophosphate. This allowed us to provide a mathematical estimation of the shifts depending on caries activity and exogenous modulation. It was shown that only for the component *parallel β-strands* in the biofilm amide profile, a statistically valid (*p* < 0.05) change in its percentage content (weight) in the presence of a cariogenic situation in the oral cavity was registered, while no significant differences were observed between patients in the normal state and those with carious pathology before and after the application of the modulator. The change in vibration frequency and percentage content of *parallel β-strands* in the spectra of the dental biofilms was a consequence of the presence of cariogenic mutans streptococci in the film, as well as of the products of their metabolism—glucan polymers.

A predictive-valid and spectroscopic-significant marker indicating the development of carious pathology in the oral cavity can be the ratio $\frac{\alpha -helix}{\beta -strands}$ in the amide profile.

The results obtained in this work suggest that the technique we used could allow the spectroscopic diagnosis of changes (shifts) in the oral microbiome associated with the development of a carious process in the oral cavity as well as become a basis for the choice of optimal therapeutic ways to treat caries based on prevention measures directed at the recovery of the microflora in the oral cavity of a patient.

## Figures and Tables

**Table 1:** Parameters (frequencies (wavenumbers), FWHM and integral intensities) of the components of the secondary structure of proteins contained in dental biofilm, determined by fitting the IR spectral profiles of the amide I and amide II bands.

Component	Assignment	I Stage		II Stage	
		Normal	Cariogenic	Normal	Cariogenic
		Wavenumber, cm^−1^ FWHM, cm^−1^ Integ. Int., cm^−2^	Wavenumber, cm^−1^ FWHM, cm^−1^ Integ. Int., cm^−2^	Wavenumber, cm^−1^ FWHM, cm^−1^ Integ. Int., cm^−2^	Wavenumber, cm^−1^ FWHM, cm^−1^ Integ. Int., cm^−2^
XV	ν(CN), δ(CH), δ(NH) [39,46]	1513.3 (±0.9) 7.8 (±0.1) 0.09 (±0.00(2))	1510.0 (±0.7) 21.5 (±0.4) 1.16 (±0.04)	1510.9 (±0.8) 25.8 (±0.6) 3.33 (±0.15)	1510.0 (±0.9) 22.6 (±0.5) 2.33 (±0.10)
XIV	*β-sheet* [30] δ_s_(NH^+^_3_) [39,46] Mutans streptococci [39]	1525.9 (±0.8) 17.4 (±0.3) 0.94 (±0.03)	1526.0 (±0.8) 23.4 (±0.7) 4.13 (±0.24)	1524.2 (±1.0) 20.3 (±0.7) 3.43 (±0.23)	1526.0 (±1.1) 22.0 (±0.7) 4.74 (±0.30)
XIII	*α-helix* [30] δ(N–H) [39,43]	1544.8 (±0.9) 25.8 (±0.6) 5.60 (±0.27)	1544.4 (±0.9) 27.0 (±1.1) 9.32 (±0.74)	1542.0 (±0.9) 26.0 (±1.1) 10.83 (±0.90)	1542.4 (±0.9) 25.5 (±1.0) 8.44 (±0.68)
XII	δ(N–H) [39,43,46]	1561.4 (±0.8) 22.0 (±0.7) 5.06 (±0.31)	1560.0 (±0.8) 22.7 (±1.0) 6.41 (±0.56)	1555.5 (±0.9) 17.7 (±0.8) 3.89 (±0.34)	1554.3 (±0.9) 21.3 (±0.9) 4.34 (±0.37)
		1570.6 (±0.7) 17.7 (±0.6) 2.88 (±0.19)	1572.5 (±0.7) 20.0 (±0.9) 6.22 (±0.56)	1566.3 (±0.8) 18.0 (±0.8) 5.31 (±0.45)	1567.0 (±0.8) 21.4 (±0.9) 6.86 (±0.58)
		1581.1 (±0.8) 17.8 (±0.6) 4.61 (±0.32)	1584.5 (±0.6) 17.7 (±0.8) 4.94 (±0.42)	1575.9 (±0.9) 16.5 (±0.6) 2.33 (±0.18)	1578.0 (±0.8) 19.0 (±0.8) 2.94 (±0.23)
XI	δ(NH_2_), amine [39,43,46]	1595.0 (±0.7) 20.8 (±0.7) 7.68 (±0.55)	1596.5 (±0.8) 17.4 (±0.7) 5.45 (±0.44)	1587.6 (±1.0) 24.0 (±0.9) 8.32 (±0.61)	1590.7 (±0.9) 24.0 (±0.9) 8.22 (±0.62)
X	Amino acid side chain [36] Intermolecular *β-sheet* [30]	1609.6 (±0.7) 18.2 (±0.7) 5.46 (±0.41)	1609.0 (±0.6) 16.1 (±0.7) 5.48 (±0.46)	1609.0 (±0.9) 24.0 (±1.0) 9.3 (±0.76)	1609.5 (±0.6) 22.0 (±0.9) 7.8 (±0.63)
IX	*β-sheet* [31,47,48,49], *β-turn* [43]	1621.5 (±0.6) 18.3 (±0.8) 3.81 (±0.39)	1619.3 (±0.7) 13.7 (±0.7) 4.47 (±0.43)	1620.0 (±0.7) 14.9 (±0.7) 3.10 (±0.30)	1619.3 (±0.5) 14.3 (±0.7) 2.93 (±0.27)
VIII	*Parallel β-strands* [43,48] Mutans streptococci [39,50]	1627.8 (±0.5) 23.4 (±1.1) 5.96 (±0.54)	1627.7 (±0.4) 12.8 (±0.7) 3.57 (±0.39)	1628.1 (±0.4) 14.8 (±0.8) 5.11 (±0.57)	1627.6 (±0.3) 14.2 (±0.7) 3.97 (±0.42)
VII	Irregular structure [30,47,48,49,51,52] Triple helix [43]	1639.5 (±0.3) 22.1 (±1.2) 10.21 (±1.08)	1638.8 (±0.4) 18.8 (±1.2) 12.85 (±1.70)	1638.7 (±0.3) 17.1 (±1.2) 10.71 (±1.48)	1638.1 (±0.3) 18.7 (±1.2) 10.96 (±1.41)
VI	*α-helix* [30,43,44,47,51,53]	1654.6 (±0.4) 22.1 (±1.4) 14.67 (±1.82)	1654.0 (±0.5) 18.7 (±1.4) 13.98 (±2.10)	1653.7 (±0.3) 20.6 (±1.5) 17.50 (±2.57)	1653.5 (±0.5) 21.1 (±1.5) 16.79 (±2.46)
V	*β-turn* [30,43,44,47,48]	1668.8 (±0.4) 20.4 (±1.3) 12.53 (±1.61)	1668.4 (±0.4) 18.4 (±1.2) 13.26 (±1.66)	1668.9 (±0.5) 17.8 (±1.3) 10.30 (±1.47)	1668.9 (±0.4) 19.0 (±1.3) 12.90 (±1.82)
IV	*Parallel β-strands* [43,47] *β-turn* [30,44,48,49,53]	1680.7 (±0.3) 18.7 (±1.1) 8.13 (±0.99)	1680.0 (±0.3) 15.0 (±0.9) 4.68 (±0.59)	1680.4 (±0.3) 14.1 (±0.7) 3.83 (±0.38)	1681.1 (±0.3) 16.1 (±1.0) 6.82 (±0.86)
III	*Anti-parallel β-strands* [43,49,51,53], *β-turn* [30,47,49]	1694.9 (±0.4) 24.0 (±1.3) 15.27 (±1.63)	1692.4 (±0.3) 24.0 (±1.2) 13.97 (±1.35)	1692.0 (±0.4) 21.4 (±0.7) 7.75 (±0.53)	1694.2 (±0.3) 21.0 (±1.0) 11.13 (±1.10)
II	ν(C=O): lipid esters [43,53]	1713.2 (±0.6) 21.6 (±0.8) 6.59 (±0.51)	1711.0 (±0.6) 17.3 (±0.5) 3.34 (±0.18)	1712.0 (±0.7) 18.6 (±0.4) 2.74 (±0.12)	1712.0 (±0.4) 18.8 (±0.5) 4.17 (±0.23)
		1729.0 (±0.7) 23.4 (±0.7) 2.99 (±0.17)	1725.3 (±0.7) 18.0 (±0.3) 1.53 (±0.05)	1726.8 (±0.9) 16.5 (±0.3) 1.11 (±0.04)	1727.7 (±0.6) 13.0 (±0.2) 0.73 (±0.03)
I	ν(C=O): acid esters [43,53]	1742.4 (±1.1) 55.0 (±1.2) 8.04 (±0.34)	1741.2 (±0.8) 34.7 (±0.5) 1.55 (±0.04)	1741.0 (±0.9) 29.0 (±0.5) 2.37 (±0.07)	1740.0 (±1.0) 31.3 (±0.4) 2.06 (±0.06)

## Data Availability

The data that support the findings of this study are available from the corresponding author upon reasonable request.
